# Functions of the UL51 protein during the herpesvirus life cycle

**DOI:** 10.3389/fmicb.2024.1457582

**Published:** 2024-08-26

**Authors:** Xiaolan Liu, Mingshu Wang, Anchun Cheng, Qiao Yang, Bin Tian, Xumin Ou, Di Sun, Yu He, Zhen Wu, Xinxin Zhao, Ying Wu, Shaqiu Zhang, Juan Huang, Renyong Jia, Shun Chen, Mafeng Liu, Dekang Zhu

**Affiliations:** ^1^Engineering Research Center of Southwest Animal Disease Prevention and Control Technology, Ministry of Education of the People's Republic of China, Chengdu, China; ^2^Key Laboratory of Animal Disease and Human Health of Sichuan Province, Chengdu, China; ^3^International Joint Research Center for Animal Disease Prevention and Control of Sichuan Province, Chengdu, China; ^4^Institute of Veterinary Medicine and Immunology, Sichuan Agricultural University, Chengdu, China; ^5^Research Center of Avian Disease, College of Veterinary Medicine, Sichuan Agricultural University, Chengdu, China

**Keywords:** herpesvirus, UL51, secondary envelopment, viral life cycle, pathogenicity

## Abstract

The herpesvirus UL51 protein is a multifunctional tegument protein involved in the regulation of multiple aspects of the viral life cycle. This article reviews the biological characteristics of the UL51 protein and its functions in herpesviruses, including participating in the maintenance of the viral assembly complex (cVAC) during viral assembly, affecting the production of mature viral particles and promoting primary and secondary envelopment, as well as its positive impact on viral cell-to-cell spread (CCS) through interactions with multiple viral proteins and its key role in the proliferation and pathogenicity of the virus in the later stage of infection. This paper discusses how the UL51 protein participates in the life cycle of herpesviruses and provides new ideas for further research on UL51 protein function.

## 1 Introduction

Herpesviridae is a linear double-stranded DNA virus that infects a variety of animals, including almost all vertebrates and even invertebrates (McGeoch et al., 1995). At present, more than 120 kinds of herpesviruses have been reported, the International Committee on Taxonomy of Viruses (ICTV) divided the herpesvirus family into three subfamilies: the α-herpesvirus subfamily, β-herpesvirus subfamily and γ-herpesvirus subfamily (Roizman et al., 1981). Each subfamily has many unclassified viruses. Among these viruses, α-herpesviruses have a wide range of hosts and can establish lifelong latent infections in sensory neurons (Cohen, 2020). Herpes simplex virus type 1 (HSV-1), herpes simplex virus type 2 (HSV-2), pseudorabies virus (PRV), duck plague virus (DPV), human herpesvirus-3, also known as varicella-zoster virus (VZV), bovine herpesvirus 1 (BoHV-1), and Marek’s disease virus (MDV) belong to the alpha herpesvirus subfamily (Wu et al., 2012; Kennedy et al., 2015; Kook et al., 2015; Boodhoo et al., 2016; Vallbracht et al., 2019). Human cytomegalovirus (HCMV), murine cytomegalovirus (MCMV), and human herpesvirus 6 (HHV-6) belong to the β-herpesvirus subfamily (Landolfo et al., 2003). Epstein–Barr virus (EBV) and Kaposi’s sarcoma herpesvirus (KSHV) belong to the γ-herpesvirus subfamily (Foulon, 1992).

**Figure 1:**
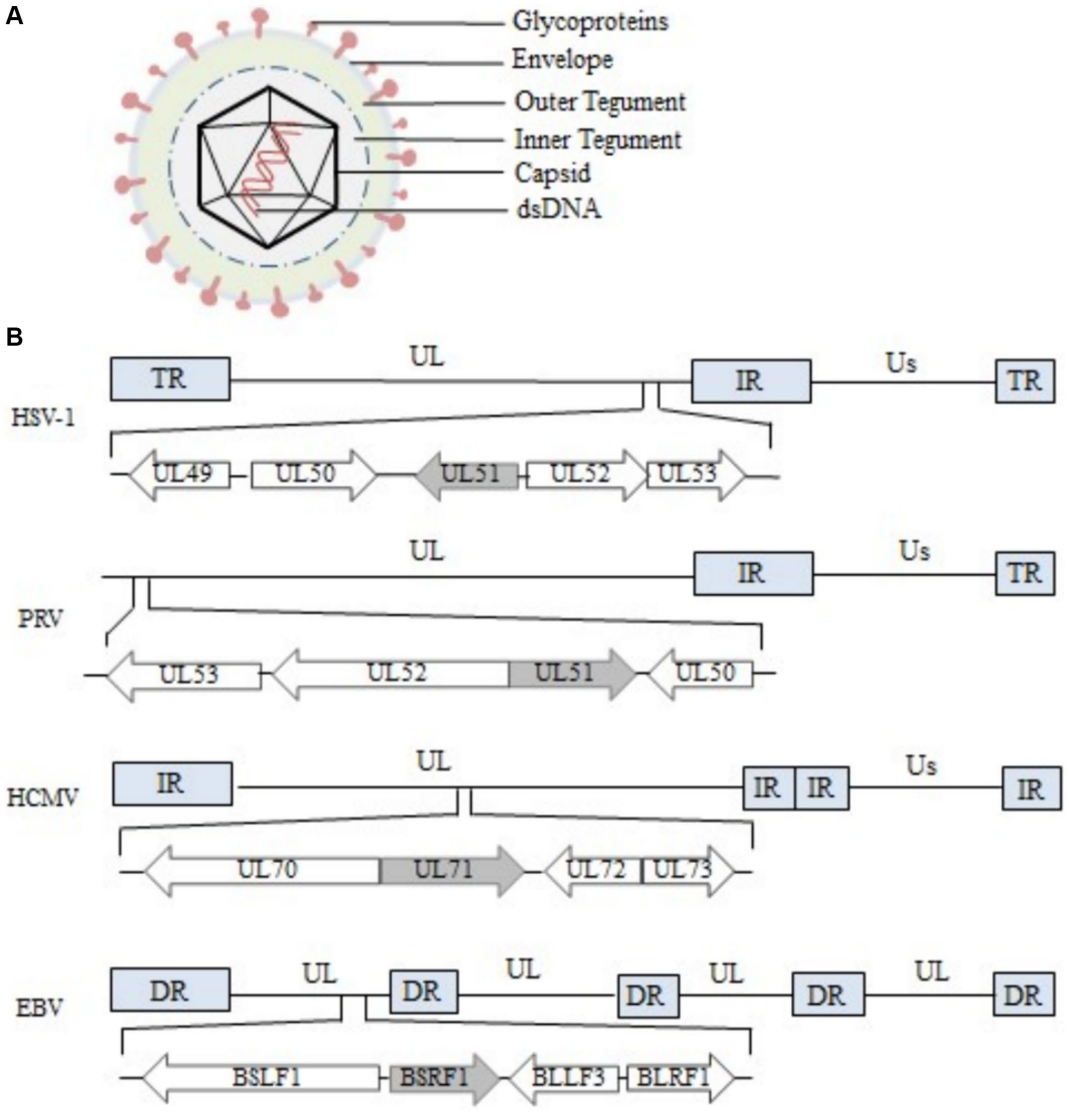
Structure of the herpesvirus genome and the region encoding the UL51 gene **(A)** Structure of HSV-1 (Liu and Zhou, 2007; Hulo et al., 2011). **(B)** The diagram shows the location and transcription direction of UL51 and its upstream and downstream genes in the HSV-1, PRV, HCMV, and EBV genomes (arrows) (Klupp et al., 2005; Nozawa et al., 2005; Sijmons et al., 2014; Majerciak et al., 2019).

Herpesvirus particles are approximately spherical, ranging in size from 120 to 300 nm, and the complete viral particle consists of four main parts from inside to outside: the genome core, the capsid, the tegument, and the vesicle (Rixon, 1993; Liu and Zhou, 2007). The linear dsDNA of the herpesvirus is covered by an icosahedral capsid (Figure 1A). The protein layer between the capsid and the envelope is the tegument, which is a unique structure of herpesviruses (Liu and Zhou, 2007). There are 26 kinds of tegument proteins found in HSV-1 (Loret et al., 2008; Kukhanova et al., 2014), which are divided into “inner” or “outer” cortical proteins according to their priority association with the capsid or envelope during the life cycle of the virus (Wolfstein et al., 2006; Radtke et al., 2010). Tegument proteins have a variety of functions, including regulating gene transcription to affect viral replication (Brulois et al., 2015; Isa et al., 2021; Turner et al., 2022; Dunn et al., 2024), acting as a bridge between viral capsid and envelope proteins, promoting the assembly of viral particles (Funk et al., 2015; Owen et al., 2015; Ivanova et al., 2016; Sucharita et al., 2023), disrupting the host innate immune response and allowing viruses to evade innate immunity (Wu et al., 2015; Fu et al., 2017; Xu et al., 2017; Deng et al., 2022; Kong et al., 2022). The herpesvirus tegument protein UL51 is involved in the regulation of multiple stages in the viral life cycle, such as the primary and secondary envelopment of the virus, the assembly and release of viral particles, and the spread of the virus between cells (Klupp et al., 2005; Nozawa et al., 2005; Dietz et al., 2018; Liang et al., 2018). This article briefly reviews the characteristics, functions, and protein interactions of the herpesvirus UL51 protein.

## 2 Characteristics of the UL51 gene and its encoded protein

### 2.1 UL51 gene characteristics

The herpesvirus genome is covalently linked by a unique long region (UL) and a unique short region (US). There are repetitive sequences at both ends of each unique region, including terminal repeat (TR) sequences, internal repeat (IR) sequences and direct repeat (DR) sequences (McGeoch et al., 1988, 1991). The UL51 gene is arranged in the following two ways in α-herpesvirus genomes: in the HSV-1 genome, it is located on the right end of the UL region (Barker and Roizman, 1990; Lee et al., 2000; Nozawa et al., 2005), the same arrangement is seen in the HSV-2, MDV-1 and MDV-2 genome (Dolan et al., 1998), and in the PRV genome, it is located on the left end of the UL region (Klupp et al., 2004, 2005), the same arrangement is seen in the VZV, EHV-1, BoHV-1, BoHV-5, ILTV, and DPV genomes (Davison and Scott, 1986; Delhon et al., 2003; Li et al., 2009). In all cases, UL51 is arranged in a head-to-head manner with the UL52 gene at the 5′ end of the genome (Ziemann et al., 1998; Klupp et al., 2004). Due to the large differences in genome arrangement among members of the herpesvirus-β and herpesvirus-γ subfamilies, there are also differences in the arrangement of the genes homologous to the α-herpesvirus UL51 gene in these two subfamilies. For example, the UL71 gene of the β-herpesvirus HCMV is arranged in a tail-to-tail manner with the UL72 gene at the 5′ end (Sijmons et al., 2014), and the BSRF1 gene and BLLF3 gene of the γ-herpesvirus EBV are also arranged in a tail-to-tail manner in the genome (Majerciak et al., 2019) (Figure 1B).

### 2.2 Types of UL51 genes

In herpesviruses, the transcription of viral genes is carried out in an orderly manner in a cascade manner, and gene expression is highly sequential (Mahmoudian et al., 2012; Dunn et al., 2024). The cascade includes three stages: the immediate early gene (IE) stage, also known as the α gene stage; the early gene (E) stage, also known as the β gene stage; the late gene (L) stage, also known as the γ gene stage (Honess and Roizman, 1974). The γ gene stage can be divided into the γ1 (partially dependent on viral DNA synthesis) and γ2 (highly dependent on viral DNA synthesis) stages (Jones and Roizman, 1979; Mahmoudian et al., 2012; Liu et al., 2015). The HSV-1 UL51 gene belongs to the γ2 class, and its expression is strictly dependent on the synthesis of viral DNA (Daikoku et al., 1998). The expression of the UL51 gene of DPV is also highly dependent on viral DNA synthesis and belongs to the γ2 class (Shen C. J. et al., 2009). The BoHV-1 UL51 gene belongs to the γ1 class, and its transcription partly depends on the synthesis of viral DNA (Hamel et al., 2002). In addition, Baer et al. analyzed the DNA sequence and encoded proteins in the EBV-B95-8 strain and found that the BSRF1 gene is a member of the γ class (Baer et al., 1984), indicating that different types of herpesvirus UL51 genes are transcribed in different ways but that they still belong to the γ class (Table 1).

**Table 1:** Characterization of the herpesvirus UL51 gene and its homologous proteins.

Subfamily	Virus	Gene	Number of amino acids	Protein molecular weight (kDa)	Gene type	References
Alphaher-pesvirinae	HSV-1	UL51	244	30	γ2	Daikoku et al. (1998)
	VZV	ORF7	259	29	ND	Selariu et al. (2012)
	PRV	UL51	236	30	ND	Lenk et al. (1997)
	ILTV	UL51	229	25	α/γ	Mahmoudian et al. (2012)
	BoHV-1	UL51	243	28	γ1	Hamel et al. (2002)
	DPV	UL51	252	29	γ2	Shen C. J. et al. (2009); Shen C. et al. (2009)
Betaher-pesvirinae	HCMV	UL71	361	55	γ	Meissner et al. (2012)
Gammaher-pesvirinae	EBV	BSRF-1	218	/	γ	Baer et al. (1984)
	KSHV	ORF55	200	/	γ	Liang et al. (2018)

### 2.3 Characteristics of the protein encoded by the UL51 gene

**Figure 2:**
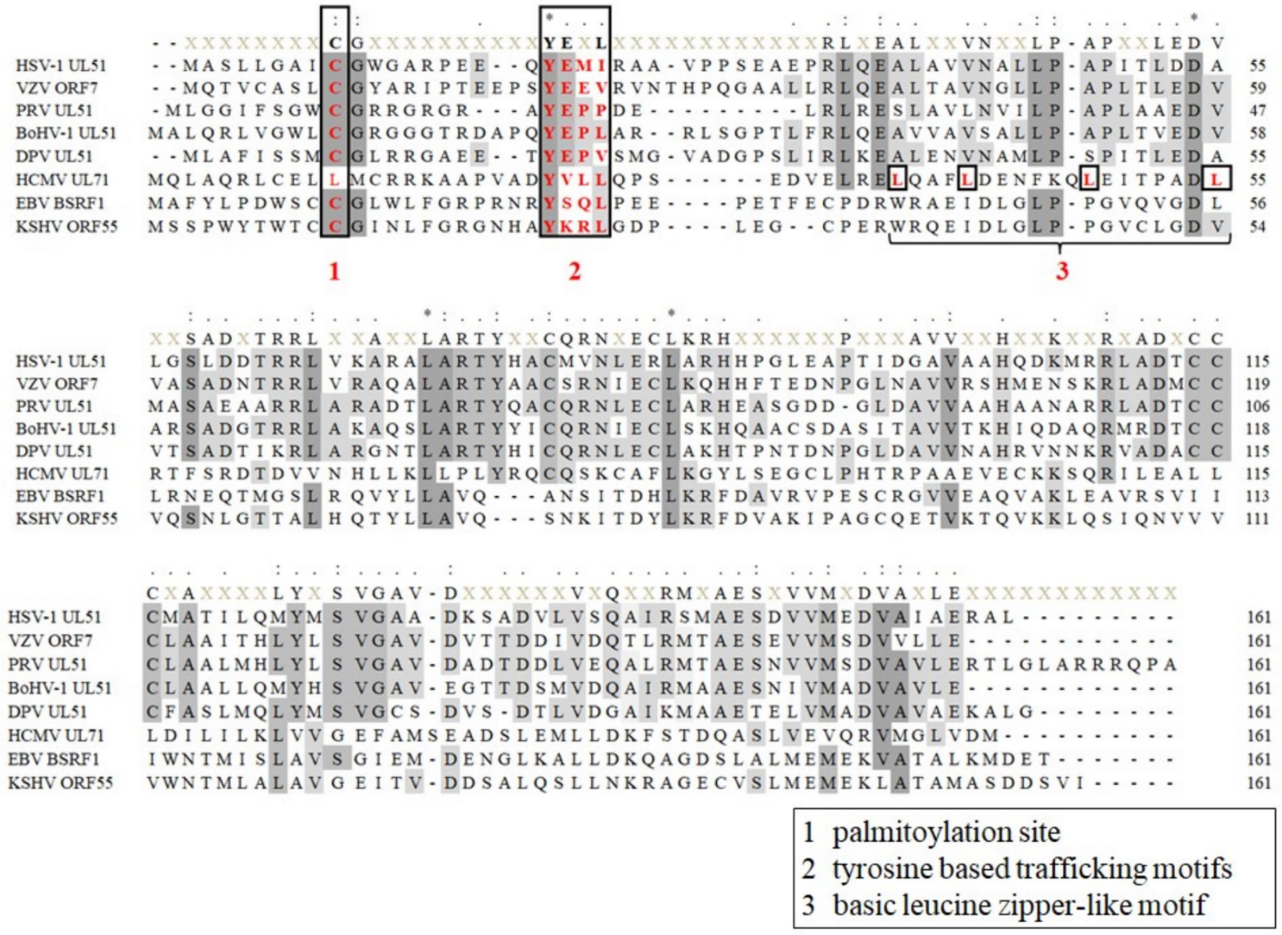
Sequence alignment of herpesvirus UL51 homologous protein N-terminal (1-161aa). (1) Palmitoylation site of HSV-1 UL51, and KSHV ORF55 proteins (Nozawa et al., 2003; Zhou et al., 2024); (2) tyrosine-based trafficking motifs in HSV-1 UL51 and HCMV UL71 proteins (Roller et al., 2014; Dietz et al., 2018); and (3) basic leucine zipper-like motif in HCMV UL71 protein (Meissner et al., 2012). Sequences of UL51 protein are collected from the complete genomes from HSV-1 (GenBank: NC 001806.2), VZV (GenBank: NC 001348.1), PRV (GenBank: MZ 219273.1), BoHV-1 (GenBank: MH 791338.1), DPV (GenBank: JQ 647509.1), HCMV (GenBank: AH 013698.2), EBV (GenBank: NC 009334.1), and KSHV (GenBank: NC 009333.1) and are aligned using MEGA 7.0 software (Kumar et al., 2016).

The UL51 protein and its homologs in herpesviruses are viral tegument proteins. The homologous proteins are relatively conserved throughout the herpesvirus subfamily and contain some conserved functional motifs (Figure 2). For example, the conserved cysteine site at the N-terminus of UL51 protein is reported to be a palmitoylation site, which is essential for protein localization and the production of infectious virus particles (Nozawa et al., 2003, 2005; Zhou et al., 2024). There is also a conserved tyrosine-based trafficking motif YXXΦ (X stands for any amino acid; Φ stands for an amino acid with a bulky hydrophobic side chain) at the N-terminus of UL51 protein, which has been shown to be involved in receptor internalization from the plasma membrane (Trowbridge et al., 1993) and protein targeting to lysosomes (Marks et al., 1995), the basolateral surface of polarized cells (Matter and Mellman, 1994), and the trans-Golgi network (TGN) (Humphrey et al., 1993). Other viral proteins in herpesvirus also contain YXXΦ motifs, such as the cytoplasmic domains of gE and gB (Alconada et al., 1999; Beitia Ortiz de Zarate et al., 2004), which play a key role in the spread of the virus. In addition to the conserved motif, there are other functional motifs, such as the basic leucine zipper-like motif of UL71 protein (Meissner et al., 2012) and the C-terminal tetralysine motif reported in HCMV (Read et al., 2019), which are beneficial to the secondary envelope process of HCMV. There are other differential motifs in the N-terminal (1–161 aa) region of the herpesvirus UL51 protein (Figure 2). Previous studies have demonstrated that Leu-110, Ile-119, and Tyr-123 in putative UL51 α-helix VI as the residues required for UL51 interaction with UL14 in HSV-1-infected cells (Oda et al., 2016). Homologs of UL7 and UL51 proteins can be identified in α-, β-, and γ-herpesviruses, although the UL51 protein lacks significant sequence similarity with homologous proteins. The interaction between UL7 and UL51 homologs is conserved across all three families of herpesviruses. Butt predicts the secondary structure of UL7 and UL51 protein homologs from representative human α-, β-, and γ-herpesviruses. It is found that the regions of pUL7 and pUL51 α-helix and β-sheet observed in the pUL7:pUL51 (8–142) core heterodimer structure, and the predicted UL7 and UL51 protein secondary structural elements are largely conserved across herpesvirus families. It is also found that UL7 and UL51 protein residues 41–142 assemble to form a heterodimeric “core” complex and that recruitment of the additional UL51 protein molecule in the native heterotrimeric complex is mediated by the N-terminal region (residues 8–40) of the UL51 protein (Butt et al., 2020).

The size of the UL51 protein varies among herpesviruses. For example, the UL51 protein exists as 27, 29, and 30 kDa phosphorylated proteins in HSV-1-infected cells (Daikoku et al., 1998). The molecular weight of the UL51 protein in DPV is approximately 34 kDa (Shen C. et al., 2009). The molecular weight of the PRV UL51 gene product is 30 kDa (Lenk et al., 1997). In BoHV-1-infected cells, the UL51 gene product is a protein with a molecular weight of 28 kDa (Hamel et al., 2002), and in VZV, the molecular weight of the protein encoded by ORF7 is 29 kDa (Selariu et al., 2012). The molecular weight of the UL51 protein in cells infected with the abovementioned herpesviruses is greater than the molecular weight predicted by software (Table 1), which may be due to posttranslational modification or differences in amino acid composition. Hydrophobic amino acids such as alanine (13.9%), leucine (7.9%), and proline (6.7%) are relatively abundant in the UL51 protein (Nozawa et al., 2003). The molecular weight of the UL71 protein in the β-herpesvirus HCMV is approximately 55 kDa, which may be due to the ability of its leucine zipper motif to dimerize via the parallel curling of helical motifs (Meissner et al., 2012).

## 3 Localization of the UL51 protein

The varying subcellular localizations of tegument proteins of different herpesviruses reflect their different functions. The UL51 protein has a conserved affinity for the cell membrane structure, especially for the Golgi membrane. In HSV-1-infected cells, the UL51 protein is mainly localized in the paranuclear region, and partially co-localized with Golgi marker protein; in transfected cells, it strongly colocalizes with Golgi marker proteins such as Golgi-58 K and GM130, and the 15 amino acid residues at the N-terminus of UL51 protein are sufficient to meet this Golgi localization characteristic (Nozawa et al., 2003). In BoHV-1-infected cells, the UL51 protein is localized mainly near the nucleus of infected cells (Hamel et al., 2002). Subsequent studies using HA-labeled BoHV-1 recombinant virus infection found that UL51 protein is completely co-localized with the Golgi marker protein GM130 (Raza et al., 2016). In PRV-infected cells, the UL51 protein is colocalized with γ-Adaptin, which is specific for the Golgi adaptor complex AP-1, or with a monoclonal antibody against an unspecified Golgi antigen (Klupp et al., 2005). Similarly, the DPV UL51 protein is localized in the cytoplasm, especially in the paranuclear region (Shen C. et al., 2009). In VZV, the ORF7 protein is located in the TGN of infected cells and forms a complex with the ORF35 protein in the TGN (Selariu et al., 2012; Wang et al., 2017). These results indicate that UL51 gene products in HSV-1, VZV, BoHV-1, PRV, and DPV are localized mainly in the cytoplasm of infected cells, and the UL51 protein of most viruses is co-localized with the Golgi marker protein, suggesting that these homologous proteins of UL51 may have similar functions. In other herpesvirus infected cells, HCMV UL71 protein is co-localized with the trans-Golgi marker protein golgin-97 in the cytoplasm and accumulate in the Golgi chamber-derived cytoplasmic virus assembly complex (cVAC) during infection (Womack and Shenk, 2010; Read et al., 2019). In addition, the EBV BSRF1 protein also colocalizes with the Golgi marker protein Giantin in the cytoplasm (Yanagi et al., 2019). Thus, in most herpesviruses, the UL51 protein is located in the Golgi apparatus. Studies have reported that the localization of the HSV-1 UL51 protein in the Golgi apparatus is mainly caused by the palmitoylation of the N-terminal 9th cysteine (Nozawa et al., 2003). This conserved palmitoylated cysteine does not exist in the MDV UL51 protein. The MDV UL51 protein is localized to mitochondria in transfected cells and to the Golgi apparatus in infected cells, indicating that the Golgi localization of the UL51 protein and its homologous proteins is conserved but is not entirely dependent on the protein itself and may also be influenced by other factors (Pasdeloup et al., 2023).

## 4 Function of UL51 in the viral life cycle

### 4.1 The life cycle of herpesviruses

**Figure 3:**
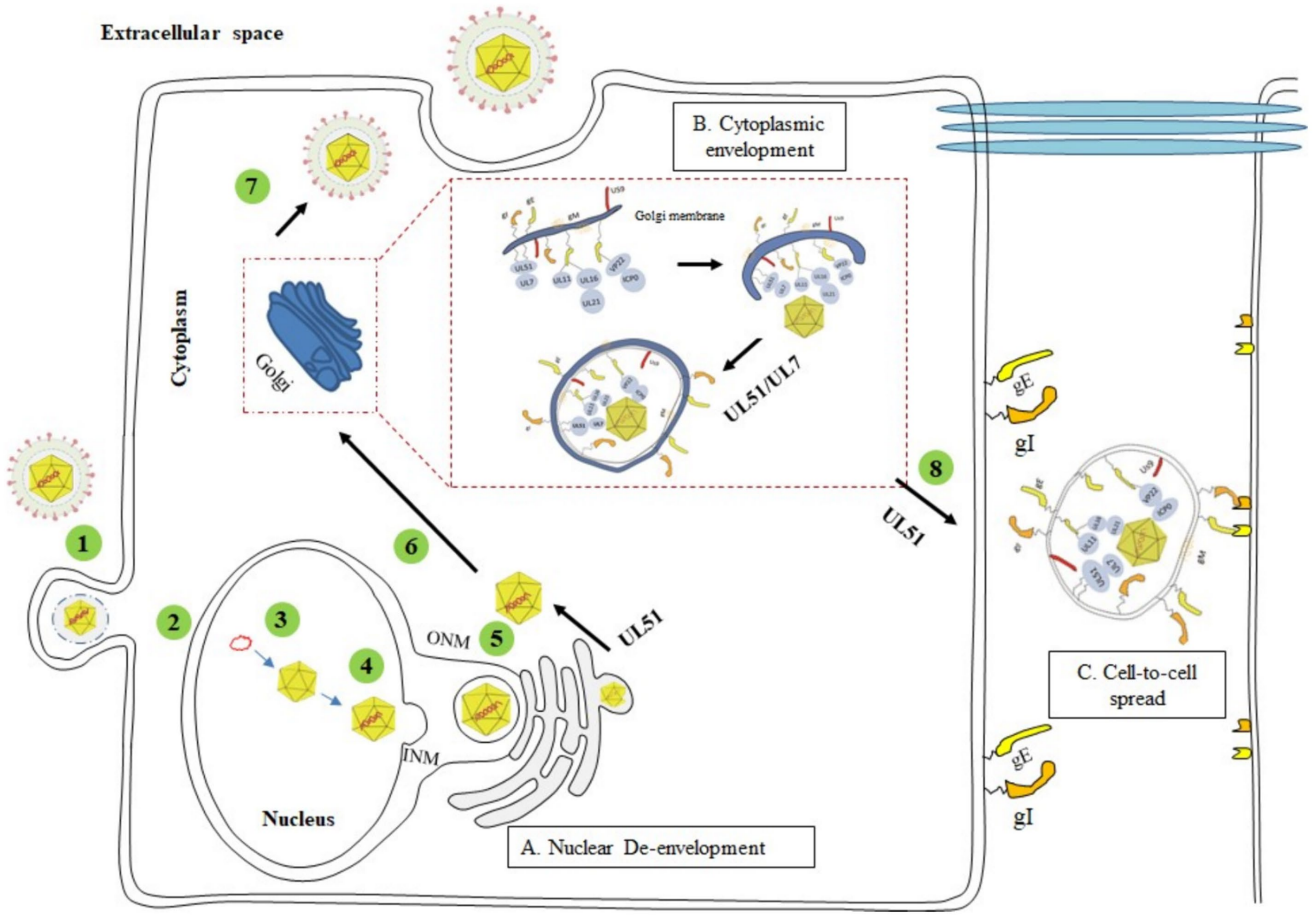
The complete process of the herpesvirus life cycle (1) Viral entry into the host cell. (2) Unenveloped viral particles enter the nucleus. (3) Nucleic acid replication and cyclization. (4) Assembly of the nucleocapsid. (5) Primary envelopment (nuclear egress) and de-envelopment. (6) Secondary envelopment. (7) Maturation and release of virions. (8) CCS of virus particles. **(A)** The UL51 protein acts at a post-inner nuclear membrane envelopment step, possibly during the outer nuclear membrane de-envelopment process (Dogrammatzis et al., 2020); **(B)** UL51 and UL7 proteins form a stable heterodimer complex on the Golgi membrane, which interacts with the capsid protein and acts as a molecular tag of the Golgi, binding the nucleocapsid to the Golgi membrane embedded with glycoproteins to promote the maturation of the virus (Nozawa et al., 2003; Roller and Fetters, 2015; He et al., 2020); **(C)** HSV-1 UL51 protein promotes the transfer of viral particles to the extracellular junction of gE/gI and binds to the receptors of adjacent cells (Roller et al., 2014; Feutz et al., 2019). Adapted from the literature (Chouljenko et al., 2012; Roller et al., 2014; Dogrammatzis et al., 2020).

Slightly differences in the mechanisms by which different herpesviruses infect host cells. α-herpesviruses (such as HSV-1, VZV, and PRV) have a shorter replication cycle and establish latent infection in the sensory ganglia of the host (Kluge and Maré, 1974; Cohen, 2020). Compared with α-herpesvirus members, β-herpesviruses (such as HCMV) and γ-herpesviruses (such as EBV and KSHV) establish latency in non-neuronal monocytes and lymphoid cells (B cells and T cells) (Chandran, 2010; Chen et al., 2022), respectively, and have longer replication cycles in cell culture (Sucharita et al., 2023). The herpesviruses of the three subfamilies also have similar life cycles (Ahmad and Wilson, 2020; Sucharita et al., 2023). Herpesvirus infection begins with entry into host cells. Virus particles are adsorbed on the cell membrane through the interaction of envelope glycoproteins and host cell-specific receptors (Shiratori et al., 2004; Atanasiu et al., 2013). After binding to cell-surface receptors, they are internalized and transported in the cytoplasm through direct fusion of the viral envelope and the plasma membrane or through endocytosis of the plasma membrane (Eisenberg et al., 2012). Subsequently, the cortical protein of the virus particles is dissociated and released into the cytoplasm, and the capsid is transported along the tubulin to the perinuclear region (Döhner et al., 2002; Radtke et al., 2010). The capsid-related viral proteins are anchored on the nucleoporin to inject the viral genome into the nucleus (Ojala et al., 2000; Abaitua et al., 2012). The transcription and replication of the viral genome occur in the nucleus. The capsid protein synthesized in the cytoplasm enters the nucleus to form a capsid, and then the viral genome is packaged into the capsid to form a nuclear capsid (Heming et al., 2017). The nucleocapsid enters the perinuclear space through budding through the inner nuclear membrane (INM) to obtain the primary envelope. After the envelope is coated, the virus particles are fused with the outer nuclear membrane (ONM) and then transported to the cytoplasm for final maturation (Bigalke and Heldwein, 2016). Viral particles in the cytoplasm bind to cortical proteins in an orderly manner, and enter the trans-Golgi network through budding to form mature viral particles by secondary envelope coating. Finally, mature viral particles are released from infected cells through exocytosis (Johnson and Baines, 2011) (Figure 3). How the UL51 protein participates in this life cycle and its functions will be discussed in detail later.

### 4.2 Effect of UL51 on viral replication

The UL51 protein is not essential for the productive replication of viruses. HSV-1 without UL51 gene can be isolated in non-proliferating cells, and the deletion virus replicates in a slightly delayed kinetics, forming very small plaques (Nozawa et al., 2005). The deletion of the UL51 gene in PRV impairs the replication of the virus in cells and mouse models to some extent, but no significant phenotype is observed (Klupp et al., 2005). In HCMV, the UL71 stop mutant could be reconstituted on noncomplementing cells proving that UL71 protein is nonessential for virus replication in human fibroblasts (Yu et al., 2003; Schauflinger et al., 2011). Similarly, the BSRF1 gene of the γ-herpesviruses EBV is not required for replication in HEK293 cells (Yanagi et al., 2019). These results suggest that the UL51 protein is not essential for viral replication, but also varies by virus species. Similar to the HSV-1 UL51 gene, the ORF7 gene of VZV is nonessential for the replication of the virus *in vitro*, but the effect of ORF7 on the growth of VZV *in vitro* is related to the type of cell. The knockout of ORF7 has no effect on the growth of VZV in MeWo cells (Zhang et al., 2010). Compared with wild-type VZV, the deletion of ORF7 will reduce the replication of VZV in ARPE-19 cells and neural progenitor cells and severed impaired replication and neurovirulence in human neuroblastoma cell lines *in vitro*. Therefore, the ORF7 protein is the first reported determinant of VZV neurotropism (Jiang et al., 2017).

The effect of the UL51 protein on viral replication is also influenced by posttranslational modification. The HSV-1 UL51 protein has been reported to be phosphorylated. Phosphorylation of UL51 at threonine 190 (Thr-190) can be detected in all virus-infected cells (Bell et al., 2013; Kulej et al., 2017), but the phosphorylation of UL51 at this site has no effect on virus replication or pathogenesis (Kato et al., 2014). Several scholars have identified five phosphorylation sites of the UL51 protein by mass spectrometry and found that the mutation of serine 184 (Ser-184) induces the abnormal accumulation of primary envelope virus particles in the nuclear membrane space and the abnormal accumulation of secondary envelope virus particles in the cytoplasm (Kato et al., 2018). However, the effect of UL51 phosphorylation at serine 184 (Ser-184) on viral replication varies with cell type and multiplicity of infection (MOI). When HaCaT cells are infected at an MOI of 0.001, the phosphorylation of UL51 at this site is necessary for the effective replication and transmission of HSV-1, but upon infection at an MOI of 5, HSV-1 replication does not require phosphorylation of the UL51 protein (Nozawa et al., 2005; Kato et al., 2018). In conclusion, the UL51 protein is non-essential for viral replication. The effects of UL51 protein and its homologous proteins on viral replication are more reflected in their impact on viral assembly (Schauflinger et al., 2011; Butt et al., 2020, 2024), participation in secondary envelopment (Nozawa et al., 2003; Meissner et al., 2012; Oda et al., 2016; Dietz et al., 2018; Read et al., 2019) and intercellular transmission (Roller et al., 2014; Feutz et al., 2019), interaction with other proteins to affect the maturation of viral particles (Nozawa et al., 2005; Roller and Fetters, 2015). The detailed process of UL51 protein involved in the life cycle of herpesvirus is shown in Figure 3.

### 4.3 The effect of UL51 on virus assembly

Mature herpesvirus particles contain a variety of different proteins that are assembled during the formation of the particles. Capsid proteins are assembled in the nucleus, while most tegument proteins and envelope proteins are assembled in the cytoplasm. Studies have reported that the formation of tegument proteins may begin at two different sites, one on the nucleocapsid after enucleation and the other in the small vesicles formed by the Golgi apparatus (Mettenleiter, 2002, 2004). Herpesvirus infection induces the rearrangement of host organelles to form multiple dispersed assembly regions in cells, which complicates the study of herpesvirus assembly (Lv et al., 2019). Due to the similarity in virion structure, all herpesviruses might be expected to undergo similar pathways for assembly The most widely studied HSV-1 has not yet been reported to form a unitary cytoplasmic virus assembly complex (cVAC) in infected cells of epithelial origin, but HSV-1 will form a unitary cVAC in cancer cells and primary neuron cells (White et al., 2020). The center concentrates the viral structural protein and is the main site of capsid assembly. The formation and maintenance of HSV-1 cVAC is related to the complete microtubule network and the viral tegument protein UL51 (Sanchez et al., 2000; White et al., 2020). The HCMV cVAC is a spatially organized system of membranes formed around the microtubule organization center (MTOC) (Sanchez et al., 2000), and its existence is microtubule and dynein motor dependent (Buchkovich et al., 2010; Indran et al., 2010). The HCMV cVAC contains enveloping capsids and concentrates a variety of viral structural proteins and markers for host cellular membranes, including Golgi-derived, and early, recycling, and late endosome-derived membranes (Sanchez et al., 2000; Das and Pellett, 2011; White et al., 2020). HCMV cVAC formation is also dependent on specific viral proteins, including UL48, UL71, UL94, UL97, and UL103. Failure to express these proteins can lead to the inability to form a normal cVAC and inhibit the production of infectious viral particles (Prichard et al., 2005; Womack and Shenk, 2010; Das et al., 2014). Although UL51 is not required for productive replication in in cell culture, it affects the production of infectious viral particles. Deletion of the UL51 gene in HSV-1 reduces the production of infectious viral particles by 1–2 orders of magnitude (Nozawa et al., 2005; Kato et al., 2018), and similarly, in HCMV UL71 mutant strains, the extracellular virus yield is reduced by an up to 16-fold (Schauflinger et al., 2011). The HSV-1 UL51 and UL7 proteins promote virus assembly by stimulating the secondary envelope of nascent viral particles (Roller et al., 2014; Roller and Fetters, 2015; Albecka et al., 2017). Similar findings have been observed for other herpesviruses. Deletion of the UL51 or UL7 protein in PRV leads to impaired viral replication and the accumulation of immature viral particles in the cytoplasm (Fuchs et al., 2005; Klupp et al., 2005). Deletion of the ORF7 protein results in a defect in the secondary envelope of VZV (Jiang et al., 2017). The viral homolog of Bcl-2 (vBcl2) in KSHV binds to ORF55 during lysis and replication, and disruption of the interaction between vBcl2 and ORF55 reduces the assembly of KSHV particles in cells (Liang et al., 2018). Deletion of the UL71 protein in HCMV leads to defects in viral replication, which is characterized by the formation of an abnormal virus assembly compartment and secondary envelopment defects (Womack and Shenk, 2010). In the resting state and during HCMV infection, mutation of the YXXΦ motif of the UL71 protein leads to the relocalization of pUL71 to the plasma membrane and hinders the assembly of HCMV (Dietz et al., 2018).

So far, there is no definite conclusion about how the UL51 protein affects the assembly process of the virus. However, recent studies have reported that the N-terminus of the HSV-1 UL51 protein can adopt functions similar to those of members of the endosomal sorting complex required for transport (ESCRT) protein family, thus participating in the assembly process of the virus (Butt et al., 2020). The endosomal sorting complex required for transport (ESCRT) machinery functions in various cellular pathways ranging from vesicular trafficking and cytokinesis to membrane repair (Vietri et al., 2020; Isono, 2021). Many of enveloped viruses, including herpesviruses, exploit the cellular ESCRT membrane remodeling machinery by recruiting ESCRT components to sites of viral budding (Rivera-Cuevas and Carruthers, 2023). The ESCRT machinery consists of four multi-protein complexes (ESCRT-0, -I, -II and-III) plus Bro-domain containing proteins like ALIX and the AAA-ATPases vacuolar protein sorting (VPS)4A and VPS4B, together these components support membrane deformation and scission events (Christ et al., 2017; McCullough et al., 2018). Among them, the interaction of ESCRT-III subunits with VPS4 drives membrane constriction and fission, with disassembly of ESCRT-III filaments to their monomeric subunits facilitating the recycling of ESCRT proteins for further rounds of assembly (Maity et al., 2019). VPS4 is recruited to sites of ESCRT-III activity via an interaction between the N-terminal Microtubule-Interacting and Trafficking (MIT) domain of VPS4 and MIT-Interacting Motifs (MIM)s located in the C-terminal tails of ESCRT-III proteins (Wenzel et al., 2022). In addition, a conserved feature of cellular ESCRT-III components (such as CHMP4B) is that they can form filaments in the neck of the nascent membrane and cooperate with VPS4 to promote membrane scission (McCullough et al., 2018; Maity et al., 2019). The N-terminal 1–170 amino acid region of UL51 protein can form filaments *in vitro*, and the structural similarity between UL51 protein and ESCRT-III component CHMP4B forms ESCRT-III–like filaments (Butt et al., 2020), suggesting a direct role for UL51 protein in promoting membrane scission during virus assembly. These results provide a structural framework for understanding the role of the conserved UL7/UL51 protein complex in herpesvirus assembly (Read et al., 2019; Butt et al., 2020). Similarly, HCMV UL71 protein may also act as a component of the viral endosomal sorting complex required for transport-III (ESCRT-III) (Streck et al., 2018). In this latest study, researchers dentify a short linear motif in the C-terminal region of pUL71 with striking resemblance to the cellular Type 2 MIM (MIM2) consensus sequence present in ESCRT-III proteins including CHMP4B and CHMP6 (Kieffer et al., 2008). It is found that the 300–325 amino acid residues of UL71 protein directly bind to the MIT domain of VPS4A, confirming that the MIM2-like motif of pUL71 is a necessary condition for VPS4A to recruit to HCMV cVAC (Butt et al., 2024). Although the functional results of pUL71 recruiting VPS4A are still unclear, the presence of conserved C-terminal vMIM2 adds additional indirect evidence to support the hypothesis that pUL71 may have ESCRT-III-like activity (Kieffer et al., 2008; Butt et al., 2024). In summary, the UL51 protein promotes viral assembly by participating in the maintenance of viral assembly complexes, regulating viral budding, and affecting the production of mature viral particles.

### 4.4 UL51 promotes secondary envelopment

In HSV-1-infected cells, the progeny virus genome is packaged into a preformed capsid in the nucleus, and the nucleocapsid of the newborn progeny virus sprouts through the inner nuclear membrane (INM) into the perinuclear space between the inner nuclear membrane and the outer nuclear membrane (ONM) to form the primary envelope (Johnson and Baines, 2011; Mettenleiter et al., 2013). The nucleocapsid wrapped by the capsule fuses with the ONM and releases the nucleocapsid into the cytoplasm, which is called decoating. Subsequently, the nucleocapsid enters cytoplasmic vesicles through budding to form the final envelope (secondary envelope) (Scheinfeld, 2010). Secondary envelope formation is an important process in the life cycle of herpesviruses. This process occurs in the Golgi apparatus, early endosomes or autophagosomes (Johnson and Baines, 2011; Buckingham et al., 2016). The immature nucleocapsid enters the cytoplasm to take up many cortical proteins, and then the secondary envelope is surrounded by the Golgi apparatus. Therefore, the Golgi apparatus is widely considered the site of secondary envelope formation (Hambleton et al., 2004; Johnson and Baines, 2011). Herpesviruses produce infectious virus particles through secondary envelopment. Mature virus particles are transported to the cell membrane through vesicles derived from the Golgi membrane and released into the extracellular space by exocytosis (Figure 3). UL51-null infections exhibit enveloped virions at the perinuclear space, as opposed to enveloped virions at membranes at the TGN during WT HSV-1 infections. The membranes that encapsulate those UL51-null virions resemble nuclear membranes through electron microscopy. Such membranes are tightly wrapped around nucleocapsids and do not appear as fuzzy as the membrane of the extracellular virions (Nozawa et al., 2005). This suggests that UL51 acts at a post-inner nuclear membrane envelopment step, possibly during the outer nuclear membrane de-envelopment process (Dogrammatzis et al., 2020) (Figure 3A). In most herpesviruses, the UL51 protein is localized to the Golgi apparatus. Deletion of this protein results in the accumulation of nucleocapsids and the complete or partial absence of secondary envelopes (Klupp et al., 2005; Raza et al., 2016; Albecka et al., 2017; Jiang et al., 2017). Therefore, the UL51 protein may be involved in targeting nucleocapsids to the final coating site or in Golgi-related viral particle transport (Nozawa et al., 2003).

The herpesvirus capsid obtains an envelope during secondary envelopment, which occurs in the cVAC in the cytoplasm of the perinuclear region of infected cells (Alwine, 2012; Procter et al., 2018). The main membranes involved in the formation of the cVAC are the Golgi membrane and the endocytic membrane (Homman-Loudiyi et al., 2003; Das et al., 2007). The HCMV UL71 protein is necessary for secondary envelopment and accumulates in the cVAC during infection, and its N-terminal trafficking motif (YXXΦ) is an important region for HCMV intracellular localization and plays an important role in the secondary envelopment of the cVAC (Dietz et al., 2018). Mutations in the YXXΦ motif of the UL51 protein in HSV-1 can lead to specific defects in viral cell-to-cell spread (CCS) (Roller et al., 2014). The driving force of HCMV membrane bending during secondary envelopment and membrane rupture is still unknown. It has been reported that the budding and membrane rupture of viral particles are regulated by viral proteins (Ahlqvist and Mocarski, 2011). In HCMV-infected cells, the absence of UL71 leads to the accumulation of nucleocapsids at the cVAC, resulting in a decrease in the proportion of envelope-encapsulated capsids. Another feature of envelope defects is the formation of extended membrane compartments associated with multiple budding events. The viral capsid accumulates in the perinuclear space and in cVAC multivesicular bodies (MVBs) (Schauflinger et al., 2011). These structural changes can impair the release and spread of the virus. The HCMV UL71 protein contains an alkaline leucine zipper domain, which is involved in the oligomerization of the UL71 protein. Mutation of this motif does not change the localization of UL71 in the viral assembly complex but leads to viral growth defects and secondary envelope defects comparable to those of UL71-deleted viruses (Meissner et al., 2012). The transport of the HCMV UL71 protein from the cVAC to the cell membrane involves the endocytic transport motif containing an N-terminal tyrosine residue (Butt et al., 2020). The elimination of this motif can lead to impaired virus growth and defective secondary envelopment. Recent studies have also shown that the C-terminal tetralysine motif in UL71 is necessary for effective HCMV secondary envelopment, which further indicates the role of the UL71 protein in the process of viral capsid formation (Read et al., 2019). Therefore, Dietz proposed an intracellular transport scheme for the HCMV UL71 protein (Dietz et al., 2018). The UL71 protein is a cytoplasmic protein; it binds to the membrane of the Golgi complex and is then transported to the plasma membrane through the secretory pathway via vesicles containing viral particles or vesicles derived from the TGN (Ford et al., 2001). UL71 is recruited to the cell membrane by the N-terminal YXXΦ motif. The vesicles formed after internalization of the cell membrane fuse with the early nuclear endosomal membrane and finally reach the endocytic recycling compartment (ERC). In the ERC, proteins are transported to the TGN, and protein transport between secretory cell compartments is mediated by adaptor protein complex 1 (AP-1) and other adaptors, which are members of the phosphofurin acidic cluster sorting (PACS) family (Crump et al., 2003). At present, the adaptor protein of the UL71 protein that targets the Golgi compartment is still unclear and needs further study.

### 4.5 UL51 promotes CCS

Herpesvirus transmission in the host depends on effective viral replication and the process by which the virus is transmitted from one cell to another in the case of host immune defense. The spread of the virus between adjacent cells is the result of CCS, in which the virus is specifically transported to the binding surface of the cell and released. Most viral proteins play a key role in viral replication, and a few viral proteins have specific functions in the intercellular transmission of viruses. For instance, the envelope glycoproteins gB, gD, and gH/gL, which are associated with viral particle entry, are required for intercellular transmission (Forrester et al., 1992; Roop et al., 1993; Cai et al., 1999; Dasika and Letchworth, 1999; Uchida et al., 2013). The heterodimer complex formed by HSV-1 gE and gI is necessary for effective intercellular diffusion in the nervous system *in vivo* and is also necessary for the diffusion of cultured neurons, epithelial cells and fibroblasts *in vitro* (Johnson et al., 1988; McGraw et al., 2009; Diwaker et al., 2020). Although gE and gI play important roles in CCS, their coding genes are only present in α-herpesviruses, so they cannot play a key role in the conserved CCS pathway. This raises the question of whether CCS is regulated by conserved herpesvirus gene products. It has been reported that HSV-1 pUL34 is a key factor affecting CCS, and this gene is also the first so-called “core” herpesvirus gene that has been clearly demonstrated to play a role in CCS (Haugo et al., 2011). UL51 is a tegument protein that is not located on the surface of viral particles, so it is unlikely to bind directly to specific receptors responsible for intercellular transmission (Lenk et al., 1997). In contrast, the localization of the UL51 protein in the Golgi allows it to be involved in the secondary envelopment of the virus and to be used as a marker of transport vesicles, as it participates in the budding of viral particles. Therefore, the UL51 protein may be key for viral cell-to-cell transmission and transport. CCS mediated by the HSV-1 UL51 protein is cell dependent. UL51-deficient HSV-1 exhibits growth and release defects in Vero cells and forms plaques that are almost 100 times smaller than those formed by the UL51-Flag tag virus. In HEp-2 cells, the CCS of UL51-deficient HSV-1 is slightly impaired, and there are no significant growth or release defects (Roller et al., 2014). Although pUL51 significantly promotes CCS in different cell types, the mechanism is different to some extent. The N-terminal YXXΦ motif of the UL51 protein is essential for CCS in HEp-2 cells. Mutations in this motif cause CCS defects comparable to those caused by UL51 deletion, but the same effect is not observed in Vero cells. In HEp-2 cells, the transport of virus particles to the cell junction surface may be mediated by the binding of the AP complex to the UL51 protein. Detailed information is presented in Table 2.

**Table 2:** Summary of the growth characteristics of some mutants of pUL51 and its orthologs in different herpesviruses.

Virus	Alteration on UL51	Plaque size	Morphology of these UL51 mutants	References
HSV1 FDL51	Deletion of codons 42 to 244	−80%^b^	Reduction in mature virus particles	Nozawa et al. (2005)
HSV1 UL51Δ73–244	Deletion of codons 73 to 244	−90% on Vero	Cell-specific defects in spread	Roller et al. (2014)
VZV-7D	Complete deletion	Altered	Increase of envelope-defective particles and a decrease in intact virions	Jiang et al. (2017)
BoHV-1 UL51Δ76–232	Deletion of codons 76 to 232	−90%	The accumulation of non-enveloped capsids in the cytoplasm	Raza et al. (2016)
MDV UL51TAP	TAP fused on Ct	−35%^a^	No difference between the v TK-TAP, vUL51-TAPiSTOP	Pasdeloup et al. (2023)
MDV UL51//TAP	No alteration	NS^a^	No difference between the v TK-TAP	Pasdeloup et al. (2023)
MDV UL51ΔCt	STOP codon at pos. 161 (Δ161-249)	NO	NO	Pasdeloup et al. (2023)
PRV ΔUL51F	Deletion of codons 68 to 233	−70%	The number of non-enveloped nucleocapsids increased, and the number of extracellular mature virus particles decreased.	Klupp et al. (2005)
hCMV TBstop71	Complete deletion except 1–12	−80%	The number of non-enveloped nucleocapsids increased, and the production of viral particles decreased by up to 16 times.	Schauflinger et al. (2011)
EBVdBSRF1-stop	Complete deletion except 1–39	Not tested	The production of progeny virus decreased.	Yanagi et al. (2019)
KSHV ΔORF55	Complete deletion	Not tested	Greatly reduce KSHV progeny virion production	Zhou et al. (2024)

^a^Compared to MDV vTK-TAP; ^b^Estimated from; NS, not significant. Some inspiration for this figure was obtained from these previous articles (Pasdeloup et al., 2023).

### 4.6 UL51 interacts with other proteins to promotes viral replication

The α-herpesvirus proteins UL7 and UL51 are tegument proteins that play a role in virus assembly and CCS. HSV-1 UL51 forms a stable and direct interaction with the UL7 protein, and the two act as a complex in infected cells, thus affecting the ability of the virus to spread between cells (Roller et al., 2014). Both of these proteins are important for HSV-1 assembly and plaque formation. Many immature capsids can be found in cells infected with UL51/UL7 mutant strains. The UL51/UL7 complex can also affect CCS via local adhesion in infected cells. Focal adhesions are contact sites between the cytoplasm and the extracellular matrix and play a crucial role in cell attachment and movement. The absence of the UL51/UL7 complex leads to focal adhesion instability and decreased cell integrity (Albecka et al., 2017). The UL51 and UL7 genes are conserved in herpesviruses. The deletion of homologous proteins (UL71 for UL51 and UL103 for UL7) in HCMV can lead to defects in the assembly compartment and secondary capsule formation, resulting in the formation of smaller infection foci. He et al. reported the complex structure of the EBV tegument proteins BBRF2 and BSRF1 (homologous proteins of UL7 and UL51, respectively), proposed the mechanism by which the BBRF2-BSRF1 complex promotes viral secondary envelopment, and revealed the conserved mode of crosstalk of these tegument proteins in the α-, β-and γ-herpesvirus families. In brief, in EBV-infected cells, BSRF1 and BBRF2 form a stable heterodimer complex on the Golgi membrane and accumulate in the Golgi apparatus, after which BBRF2 binds to the MCP and BPLF1 proteins from the nucleocapsid to form a sticky mat. The accumulated BBRF2-BSRF1 complex acts as a molecular tag for the Golgi apparatus, binding unenveloped capsids exported from the nucleus to the glycoprotein-embedded Golgi membrane and ultimately promoting secondary envelopment (He et al., 2020) (Figure 3B).

The UL51 protein also interacts with UL7 and gE in infected cells. Partial elimination of UL51 or UL7 causes gE to not accumulate on the binding surface of Vero cells, and the gE-UL7-UL51 functional complex of HSV-1 is located at the cell surface junction, where gE accumulates and forms syncytia, affecting the spread of the virus. By analyzing the transmission phenotype of gE and UL51 double deletion viruses, it was found that gE and UL51 have the same transmission pathway in HaCaT cells (Feutz et al., 2019). However, compared with gE deletion viruses, UL51 deletion viruses exhibit more severe defects in intercellular transmission, indicating that the UL51 protein participates in epithelial cell transmission in a gE-independent manner (Roller et al., 2014) (Figure 3C). UL51 and UL14 form a complex in HSV-1-infected cells, which may anchor to the cell membrane and interact with the viral envelope protein gE and capsid proteins VP26 and VP19C (Lee et al., 2008; Fossum et al., 2009; Roller et al., 2014; Oda et al., 2016). Through these interactions, the nucleocapsid and cell membrane are bridged to promote secondary envelopment and intercellular transmission of the virus to achieve effective viral replication (Oda et al., 2016). In summary, herpesvirus UL51 and its interacting proteins are concentrated on the cell surface, where they affect the morphology of virus-infected cells, stabilize focal adhesions on cell membranes, and provide physical support for cells.

### 4.7 UL51 affects virus pathogenicity

The envelope proteins and tegument proteins of herpesviruses play important roles in the pathogenesis of the virus. The UL51 protein affects the pathogenicity of HSV-1 during intracranial infection in mice, and phosphorylation of the UL51 at Ser-184 is necessary for high virulence of HSV-1 during ocular infection in mice (Kato et al., 2018). The absence of the BoHV-1 UL51 protein can lead to severe viral growth defects, manifested as reduced one-step and multistep growth kinetics, decreased plaque size, and the accumulation of nonenveloped capsids in the cytoplasm of infected cells *in vivo*. After infection of rabbits by nasal drip, the UL51 deletion strain does not cause clinical symptoms, and nasal detoxification does not occur, indicating that UL51 deletion in BoHV-1 impairs viral growth *in vitro* and decreases virulence in rabbits (Raza et al., 2016). The C-terminal fusion TAP tag of the MDV UL51 protein can lead to impaired viral function, impaired viral replication *in vivo*, and significantly reduced virulence, indicating that the UL51 protein is essential for the growth of MDV *in vitro* and plays a key role in the pathogenesis of MDV infection *in vivo* (Pasdeloup et al., 2023). ORF7, a homologous protein of UL51 in VZV, has been reported to be a neurovirulent factor and can be used as a target for antiviral drugs. Studies have reported that ORF7 deletion in VZV using a stop codon mutation strategy reduces the effects of the virus on skin and nerves and reduces the risk of vaccine-related complications. Similar to the VZV vaccine strain Oka, ORF7-deleted VZV strain retains complete lymphotropicity, which allows the replication and transmission of the vaccine strain *in vivo* (Wang et al., 2022a,b). ORF7-deleted VZV can stimulate the adaptive immune system of the human body and has potential as a live vaccine. Recently, during the development of therapeutic drugs for VZV infection based on RNA interference (RNAi), it was found that recombinant ORF7-siRNA (r/si-ORF7) could inhibit VZV infection *in vitro* and *in vivo* and maintain normal skin morphology, indicating that r/si-ORF7 could be a potential candidate for the clinical treatment of VZV infection (Pei et al., 2023). Furthermore, the ORF7 protein is an ideal target for inhibiting the replication of the VZV genome. In general, according to existing research, compared with that of the wild-type virus, the virulence of UL51-deleted virus is significantly reduced, indicating that the UL51 protein may be a virulence factor of herpesviruses and play an important role in the pathogenicity of viruses.

## 5 Summary and prospects

Among the α-, β-and γ-herpesviruses, UL51 is a conserved tegument protein that plays a wide role in the life cycle of herpesviruses, affecting viral replication and regulating viral assembly and transmission. Moreover, the protein–protein interaction network in herpesviruses is dynamic, so the interaction network of the UL51 protein is very important for virus assembly and secondary envelopment. In the future, studies of conserved complexes, such as the UL51/UL7 complex, can be performed to investigate the functions of UL51 and its interaction proteins to determine whether they are related to the bridge between the capsid and the envelope. At present, there are few studies on the interaction between the UL51 protein and host proteins. It is worth exploring whether the mechanism by which host proteins are involved in virus assembly and transport can be identified by studying the interaction between pUL51 and host proteins. Therefore, an in-depth understanding of the molecular mechanism by which the UL51 protein regulates the life cycle will provide new insights for the development of treatment for herpesvirus infection via the destruction of key proteins in the life cycle of herpesvirus, thus providing a new strategy for the treatment of herpesvirus infection and resulting diseases.
